# Real time near-infrared Raman spectroscopy for the diagnosis of nasopharyngeal cancer

**DOI:** 10.18632/oncotarget.17703

**Published:** 2017-05-08

**Authors:** Lim Chwee Ming, Nagaraja Rao Gangodu, Thomas Loh, Wei Zheng, Jianfeng Wang, Kan Lin, Huang Zhiwei

**Affiliations:** ^1^ Department of Otolaryngology-Head and Neck Surgery, National University Health System, Singapore; ^2^ Division of Surgical Oncology, National Cancer Institute of Singapore, Singapore; ^3^ Optical Bioimaging Laboratory, Department of Biomedical Engineering, Faculty of Engineering, National University of Singapore, Singapore; ^4^ Department of Medicine, Yong Loo Lin School of Medicine, National University of Singapore and National University Health System, Singapore

**Keywords:** Raman spectroscopy, nasopharyngeal cancer, real time imaging, PLS-DA model, surveillance

## Abstract

Near-infrared (NIR) Raman spectroscopy has been investigated as a tool to differentiate nasopharyngeal cancer (NPC) from normal nasopharyngeal tissue in an ex-vivo setting. Recently, we have miniaturized the fiber-optic Raman probe to investigate its utility in real time in-vivo surveillance of NPC patients. A posterior probability model using partial linear square (PLS) mathematical technique was constructed to verify the sensitivity and specificity of Raman spectroscopy in diagnosing NPC from post-irradiated and normal tissue using a diagnostic algorithm from three significant latent variables. NIR-Raman signals of 135 sites were measured from 79 patients with either newly diagnosed NPC (*N* = 12), post irradiated nasopharynx (*N* = 37) and normal nasopharynx (*N* = 30). The mean Raman spectra peaks identified differences at several Raman peaks at 853 cm^−1^, 940 cm^−1^, 1078 cm^−1^, 1335 cm^−1^, 1554 cm^−1^, 2885 cm^−1^ and 2940 cm^−1^ in the three different nasopharyngeal conditions. The sensitivity and specificity of distinguishing Raman signatures among normal nasopharynx versus NPC and post-irradiated nasopharynx versus NPC were 91% and 95%; and 77% and 96% respectively. Real time near-infrared Raman spectroscopy has a high specificity in distinguishing malignant from normal nasopharyngeal tissue *in vivo*, and may be investigated as a novel non-invasive surveillance tool in patients with nasopharyngeal cancer.

## INTRODUCTION

Non-keratinizing nasopharyngeal cancer (NPC) is a cancer that originates from the nasal epithelium of nasopharynx and it is often difficult to detect in the early stage due to the deep anatomical location. It is a common cancer in Asia where the incidence reaches its peak in countries such as Hong Kong, Southern China and Singapore. In Singapore, it is the 8th most common cancer among males [1] and carries with it risk of loco-regional recurrence of up to 20% following definitive chemo-radiotherapy [2].

Early detection of local recurrence in NPC is pivotal towards successful surgical salvage, which has been shown to improve survival [3]. However, the challenge lies in having a reliable and accurate method of detecting early recurrence. Currently, the most reliable way of detecting nasopharyngeal recurrence is endoscopic examination of the nasopharynx, although the morphology of early recurrences is unknown. Most cases of recurrences are detected as a recurrent mass or ulcer in the nasopharynx. These recurrences are sometimes not surgically salvageable due to local extension of the tumor to critical structures such as the internal carotid artery, skull base and optic nerve that are in close proximity to the nasopharynx. Therefore, identifying early morphologic changes preceding the development of these clinically obvious recurrences will allow prompt surgical salvage of these recurrences; and hence improve clinical outcomes of NPC patients.

Raman spectroscopy is a unique optical technique, which utilizes inelastic scattering of light from tissue to characterize its molecules and tissue composition. This system has been well studied in gastric cancer in diagnosing dysplasia and premalignant condition [4–6]. Lau DP et al. reported a preliminary analysis on differentiating nasopharyngeal cancer from normal tissue *ex vivo* using Raman spectroscopy [7]. However, the data was not optimized as it only covered a narrow spectral window of 950–1650 cm^−1^ with no statistical significance. Recently, Huang's group [8] has developed a rapid fiber-optic NIR Raman spectroscopy system for better characterization of tissue Raman signals *in vivo*, and miniaturizing the probe size to 1.8 mm; which allows clinicians to obtain a more reliable Raman signals in tight anatomical confines in the nasopharynx.

Putting this together, we aim to test this new NIR Raman spectroscopy method in detecting distinctive molecular signatures specific to normal healthy nasopharynx, post-irradiated nasopharynx and NPC tissue. The clinical relevance of this study is to investigate if this tool may be employed in the surveillance of NPC patients; with the overarching goal to detect early Raman signals which precede the development of clinically apparent local recurrence in the nasopharynx of these patients.

## RESULTS

**Figure 1:**
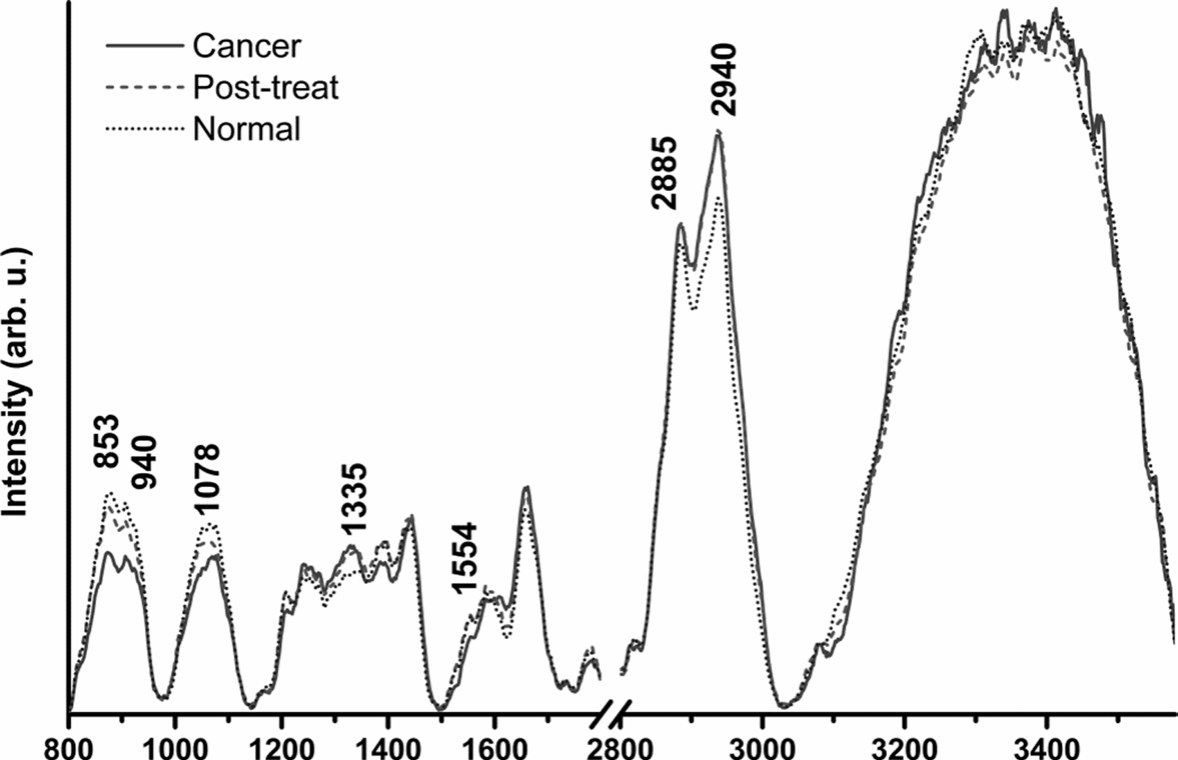
Raman Spectra peaks for patients with normal nasopharynx, post irradiated nasopharynx and nasopharyngeal cancer

Raman signatures (*N* = 135) were obtained from 79 patients (12 NPC; 30 normal patients and 37 post-irradiated) and the patients' characteristics are summarized in Table 1. When the cumulated Raman spectra peaks of these patients were analyzed, distinct differences is mean amplitudes in Raman peaks were identified at 853 cm^−1^, 940 cm^−1^, 1078 cm^−1^, 1335 cm^−1^, 1554 cm^−1^, 2885 cm^−1^ and 2940 cm^−1^among patients with NPC, normal nasopharynx and post-irradiated nasopharynx (Figure 1).

**Table 1:** Summary of patient's characteristics

Category	Normal Nasopharynx	Post-Irradiated Nasopharynx	Nasopharyngeal cancer
**Number of patients (*n* = 79)**	30	37	12
**Number of collected sites data (*n* = 135)**	42	71	22
**Mean Age (S.D.)**	54.4 (18.9)	52.9 (12.3)	52.6 (11.7)
**Gender**	23 Males/ 7 Females	27 Males/ 10 females	6 Males/ 6 Females
**Stage**	N/A	N/A	I–IV

**Figure 2:**
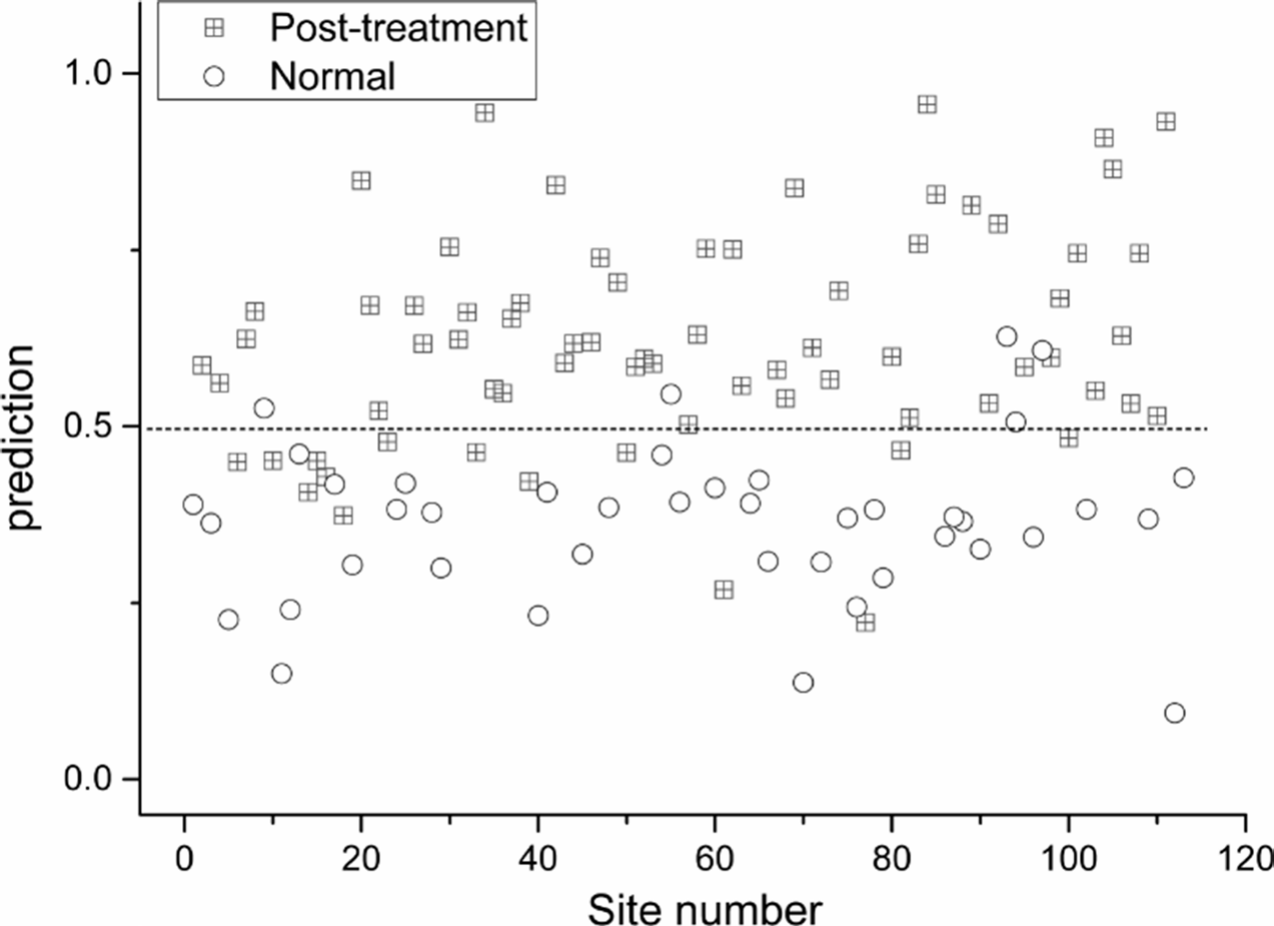
Scatter plot of the posterior probability model using partial linear square technique of patients with normal nasopharynx versus post-irradiated nasopharynx (sensitivity 80%; specificity 88%)

**Figure 3:**
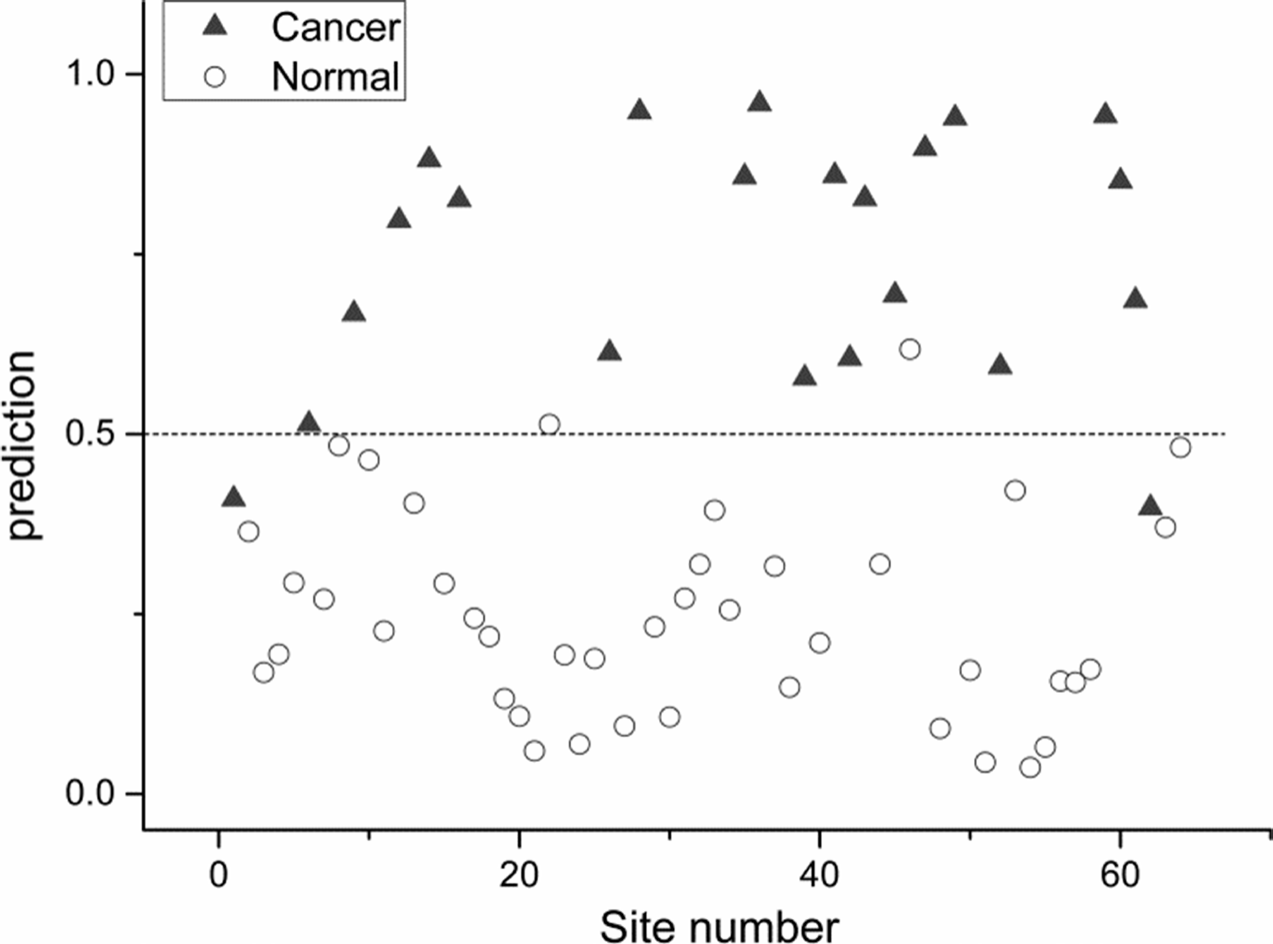
Scatter plot of the posterior probability model using partial linear square technique of patients with normal nasopharynx versus nasopharyngeal cancer (sensitivity 91%; specificity 95%)

**Figure 4:**
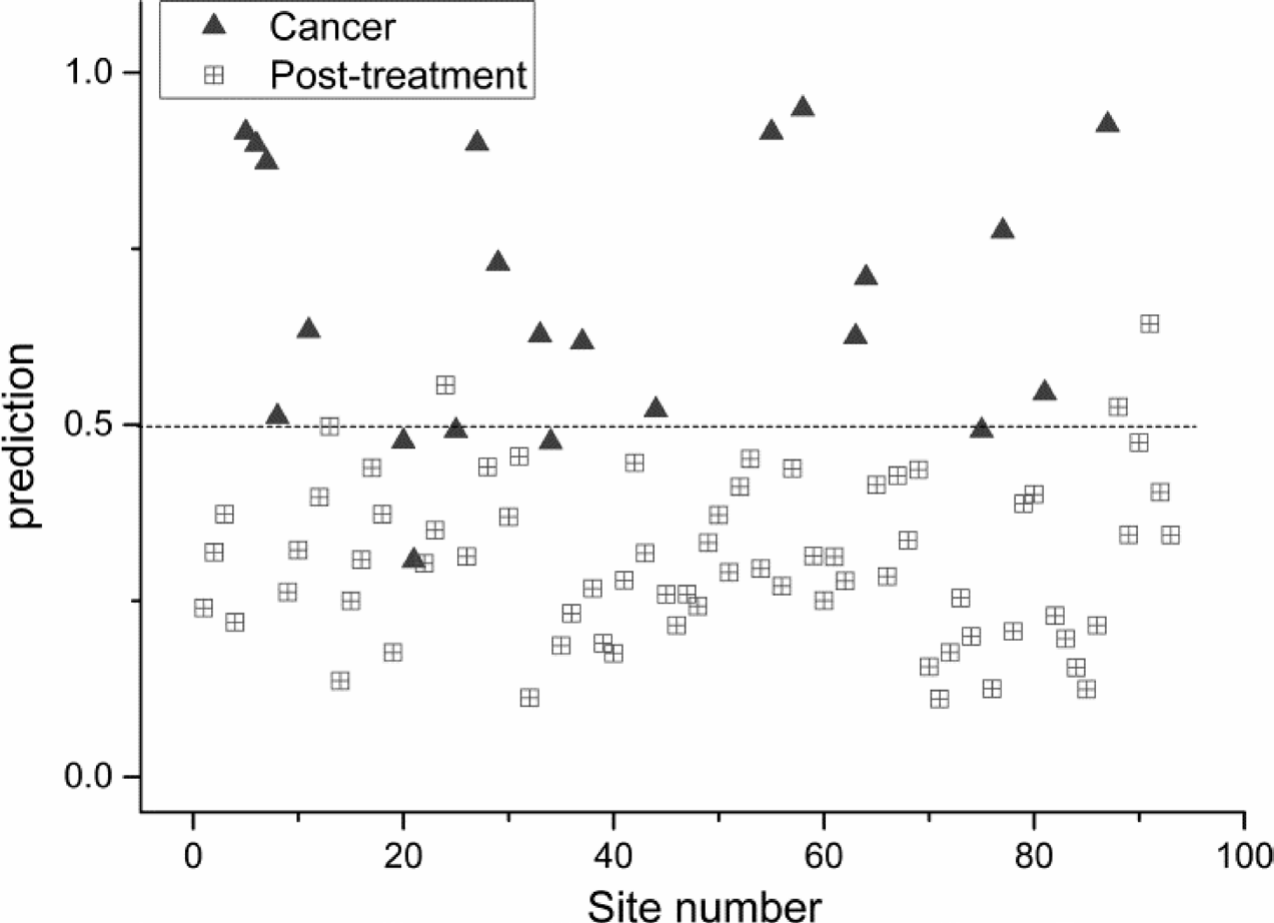
Scatter plot of the posterior probability model using partial linear square technique of patients with post-irradiated nasopharynx versus nasopharyngeal cancer (sensitivity 77%; specificity 96%)

Using partial least squares regression method, comparison of collective Raman signatures between normal nasopharynx versus post-irradiated nasopharynx (Figure 2); NPC versus normal nasopharynx (Figure 3); and NPC versus post-irradiated nasopharynx were constructed (Figure 4). The corresponding sensitivity and specificity for the respective paired cohorts were post-irradiated nasopharynx versus normal nasopharynx (sensitivity 80%; specificity 88%); normal nasopharynx versus NPC (sensitivity 91%; specificity 95%); and lastly between post irradiated nasopharynx versus NPC(sensitivity 77%; specificity 96%).

## DISCUSSION

Utilizing Raman spectroscopy in cancer diagnostics has gained momentum over the past decade. This technology has been used in the differentiating normal and cancerous tissues in lung cancer [9, 10], gastric cancer [6, 11, 12], cervical cancer [13], laryngeal and nasopharyngeal cancer [7, 14–16]. Much of this enthusiasm lies in the capability and reliability of Raman scattering signals to reveal biomedical and bio-molecular signatures that are unique to the tissue composition being evaluated and hence, provide an opportunity to distinguish different pathologies on tissues at a molecular level.

In this pilot study, we sought to examine the Raman signatures in 3 patient cohorts, namely in patients with nasopharyngeal cancer, normal nasopharynx and post-irradiated nasopharynx following radiotherapy for nasopharyngeal cancer. The main motivation in performing this pilot study is to establish if a differential Raman scattering signatures may be obtained reliably using a 1.8 mm probe, which is currently the smallest probe utilized towards Raman spectroscopy diagnostics. With this validation study, the possibility of using Raman spectroscopy may be envisaged in earlier diagnosis of local recurrence of nasopharyngeal cancer; which is usually detected late given the deep anatomical location.

Our real-time in-*vivo* test using this fiber-optic-Raman (1.8 mm) probe was successful in obtaining reliable and consistent Raman signatures among patients with nasopharyngeal cancer, post-irradiated nasopharynx and normal controls. There were several Raman spectra peaks whereby distinct Raman scattering peaks were obtained and hence, validated our hypothesis that these tissues exhibited different tissue compositions, which can be picked up by Raman spectroscopy. We have adopted the PLS algorithm in constructing our normogram data based on our previous published reports [17, 18] as well as a recent study in nasopharyngeal cancer, where the PLS algorithm was more sensitive than the PCA model in detecting malignant Raman signatures in nasopharyngeal cancer [19]. Additionally, the PLS regression method further rotates the components (latent variables (LVs)) to achieve the maximum group separation compared to the PCA model. Hence, these LVs could explain the diagnostic relevant variations rather than the significant differences in the dataset.

We believe that our study represents a step forward in the development of Raman spectroscopy in the diagnostic of nasopharyngeal cancer on several counts. Firstly, ever since the first report of using Raman spectroscopy in nasopharyngeal cancer by Lau et al. [7], all previous studies have been based on using Raman signatures taken on ex-*vivo* nasopharyngeal tissues which may result in differences in the Raman signatures due to desiccation efforts following tissue ex-plantation [15, 19, 20]. Therefore, the clinical utility of real-time molecular diagnostics using Raman spectroscopy cannot be directly translated. Secondly, to our knowledge, the1.8 mm probe size is the smallest size being used for evaluation in Raman spectroscopy. Li et al. [15] performed micro-Raman spectroscopy on tissue samples in differentiating the Raman signatures between normal nasopharyngeal tissue and nasopharyngeal cancer. The micro-Raman tissue measurements based on commercially bulky Raman microscope system are impractical for clinical Raman applications at endoscopy. Therefore, a smaller caliber probe is more desirable. Lastly, we have included an additional cohort of patients following radiotherapy of their nasopharyngeal cancer. In this regard, Raman signatures among patients with post-irradiated nasopharynx may be investigated and studied so that any Raman shifts between post-irradiated nasopharynx and nasopharyngeal cancer may be analyzed. This has translational application towards surveillance of the nasopharynx for recurrence following radiotherapy.

Our pilot study demonstrated a relatively high specificity of 95% and 96% in distinguishing normal nasopharynx versus nasopharyngeal cancer and post-irradiated nasopharynx versus nasopharyngeal cancer. With this high specificity values, we believe our *in vivo* fiber-optic Raman spectroscopy system has the potential for real time surveillance of nasopharyngeal cancer by selecting patients with “malignant” Raman signatures for further confirmation by histology. Additionally, patients with previously treated nasopharyngeal cancer who present with a normal looking nasopharynx plus a “benign” Raman signature may be more reassured of having no local disease; rather than relying on a purely normal looking nasopharyngeal finding on endoscopy.

We demonstrate for the first time that real-time near-infrared Raman spectroscopy using a 1.8 mm fiber-optic Raman probe can detect reliable and consistent Raman scattering signatures among patients with nasopharyngeal cancer, post-irradiated nasopharynx and normal nasopharynx. The high specificity in distinguishing malignant from normal nasopharyngeal tissue and post-irradiated nasopharyngeal tissue warrants investigation of this tool as a new non-invasive surveillance tool in patients with nasopharyngeal cancer.

## MATERIALS AND METHODS

Under an Institutional approved clinical protocol (National Healthcare Group DSRB; 2014/00323), prospective patients with newly diagnosed NPC, post-irradiated nasopharynx (at least 6 months following completion of radiotherapy) and normal nasopharynx were accrued in this study. Patients with nasopharyngeal cancer were biopsied proven to be cancer while patients with normal nasopharynx were identified based on normal endoscopic examination of the nasopharynx. Patients with post-irradiated nasopharynx were those who had received prior definitive radiotherapy for nasopharyngeal cancer and were disease free at the time of examination. This assessment was based endoscopic examination of the nasopharynx showing no residual tumor as well as a post-treatment magnetic resonance imaging (MRI) scan of the nasopharynx demonstrating complete resolution of their initial tumor. Any clinically suspicious areas of the nasopharynx were biopsied and were all proven to be negative of cancer. Additionally, all these patients with post-irradiated nasopharynx were followed up for at least 6 months with no evidence of recurrence.

### Detection of Raman signals of the nasopharynx

**Figure 5:**
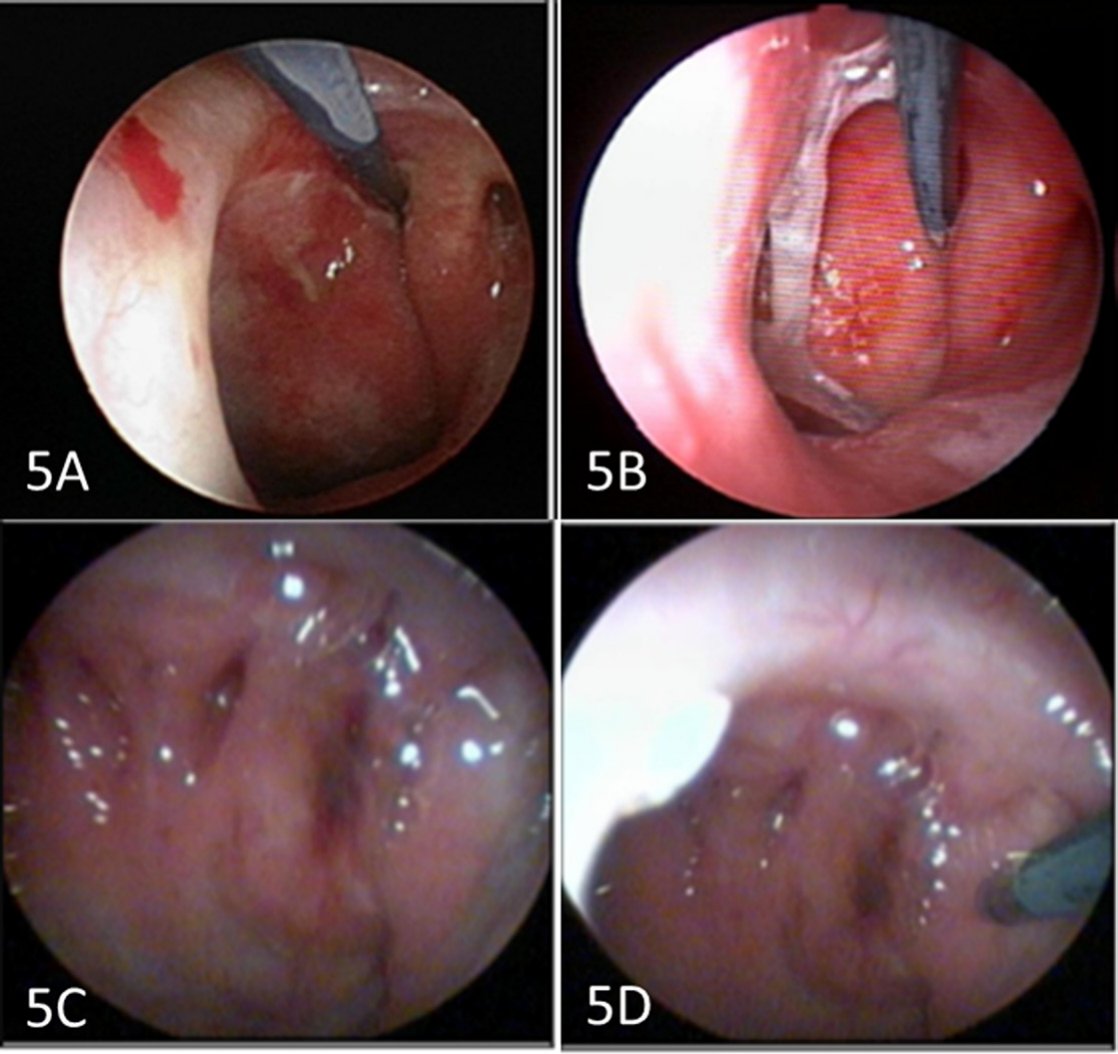
Endoscopic view of the micro-Raman probe in contact with nasopharynx- post-irradiated (**A**, **B**) and nasopharyngeal cancer (**C**, **D**).

During endoscopic examination of the nasopharynx, a1.8-mm miniature Raman probe is navigated to the nasopharynx under direct vision from the endoscope. In patients with normal and post irradiated nasopharynx, the probe is placed close to the fossa of Rosenmuller and additional measurements were performed in the midline of the nasopharynx of some patients (Figure 5). In patients with newly diagnosed (pre-treatment) NPC, the probe is positioned directly on the tumor (Figure 5). Once contact to the nasopharynx is achieved, a NIR laser is emitted at 785-nm from the probe and the back-scattered light (Raman signal) is captured by the Raman system. In this study, we have adopted a simultaneous fingerprint (FP) and high wavenumber (HW) fiber-optic Raman spectroscopy technique for real-time *in vivo* tissue Raman measurements during endoscopy. The 785-nm laser excitation power at ∼12 mW was selected as it was within the maximal permissible skin exposure limit set out for a 785-nm laser beam (American National Standards Institute). Multiple spectra (∼8–10) for each tissue site were measured with scanning times of 0.1 to 0.5 sec, which permits a rapid survey of the tissue areas. The entire process of capturing of Raman signals is typically completed within 15–20 seconds.

### Construction of the nomogram data

The NIR Raman spectra measured from in-*vivo* nasopharyngeal tissue represent a combination of weak tissue Raman signal, intense auto-fluorescence (AF) background and noise. The raw spectra are preprocessed by a third-order Savitzky-Golay smoothing filter (a window width of 3 pixels) to remove the spectral noise [8, 14, 17, 18]. In the finger-print (FP) region (800–1800 cm^−1^), a fifth-order polynomial was found to be optimal for fitting the AF background in the noise-smoothed spectrum, and this polynomial was then subtracted from the measured FP spectrum to yield the FP tissue Raman spectrum alone. In the high-wave number (HW) range (2800–3600 cm^−1^), a first-order polynomial fit was used for removing the AF background [17, 21, 22]. The FP/HW Raman spectra are then normalized over the integrated area under the FP and HW ranges to allow a better comparison of the spectral shapes and relative Raman band intensities among NPC, normal and post-irradiated tissues.

Partial least squares (PLS) (also called Projection to Latent Structure) regression is a validated method for modeling in Raman system, providing an alternate approach to PCA (Principle component analysis). PLS technique is an extension of the multiple linear regression model and a linear model specifies the (linear) relationship between a dependent (response) variable Y, and a set of predictor variables, the X's. It follows the principle of PCA, but further rotates the components (latent variables (LVs)) to achieve the maximum group separation. Hence, the LVs could explain the diagnostic relevant variations rather than the significant differences in the dataset. Many studies have constructed and validated this statistical model to predict the sensitivity and specificity of Raman system in NPC model [15, 23, 24]. Additionally, consistent and reproducible results using this statistical model have been validated in various cancers including, gastric cancer [11, 12], esophageal cancer [11, 25], colon cancer [21, 26] and NPC [14].

## Abbreviations

NPC: Nasopharyngeal carcinoma
PLS-DA: Partial Least Square- Discriminant Analysis
FP: Finger Print
HW: High Wave number
NIR: Near Infra-Red
AF: Auto florescence
